# Biomimetic Nanocarriers for Glioblastoma Therapy: Translational Advances and Strategic Challenges

**DOI:** 10.3390/cancers18182982

**Published:** 2026-09-15

**Authors:** Bhawana Jain, Sunita Sanwaria, Arti Hadap, I Made Joni, Renny Febrida, Rovina Ruslami, Irwan Purnama, Deoraj Singh, Camellia Panatarani

**Affiliations:** 1 Department of Chemical Sciences, Siddhachalam Laboratory, Raipur 493221, Chhattisgarh, India; bhawana.jain@unpad.ac.id; 2 Functional Nano Powder University Center of Excellence (FiNder U CoE), Universitas Padjadjaran, Jl. Raya Bandung-Sumedang KM 21 Jatinangor, Sumedang 45363, West Java, Indonesia; arti.hadap@nmims.edu (A.H.); imadejoni@phys.unpad.ac.id (I.M.J.); renny.febrida@unpad.ac.id (R.F.); 3 Department of Chemistry, Government Nagarjuna Post Graduate College of Science, Raipur 4920010, Chhattisgarh, India; ssanwaria@db.du.ac.in; 4 Department of Basic Science and Humanities, Mukesh Patel School of Technology Management and Engineering, SVKM’s Narsee Monjee Institute of Management Studies (NMIMS) Deemed-to-University, Mumbai 400056, Maharashtra, India; 5 Department of Physics, Universitas Padjadjaran, Jl. Raya Bandung-Sumedang KM 21 Jatinangor, Sumedang 45363, West Java, Indonesia; 6 Department of Dental Materials, Faculty of Dentistry, Universitas Padjadjaran, Jl. Raya Bandung-Sumedang KM 21 Jatinangor, Sumedang 45363, West Java, Indonesia; 7 Department of Biomedical Sciences, Faculty of Medicine, Universitas Padjadjaran, Jl. Raya Bandung-Sumedang KM 21 Jatinangor, Sumedang 45363, West Java, Indonesia; rovina.ruslami@unpad.ac.id; 8 School of Electrical Engineering, Telkom University, Jl. Telekomunikasi No.1, Citeureup, Dayeuhkolot, Bandung 40257, West Java, Indonesia; irwa010@brin.go.id; 9 National Research and Innovation Agency (BRIN), Bandung 40135, West Java, Indonesia; 10 Department of Biochemistry, Weill Cornell Medical College, New York, NY 10065, USA; drsingh@wisc.edu

**Keywords:** glioblastoma, biomaterials, immunotherapy, bioavailability, BBB penetration

## Abstract

Glioblastoma (GBM) is the most aggressive and common type of brain tumor, characterized by rapid growth and infiltration, as well as high resistance to conventional treatments. Given the persistent challenge posed by the bloodbrain barrier (BBB) to effective drug delivery, a shift in therapeutic strategies is required. Biomimetic nanocarriers (BNCs) represent a promising approach for the targeted treatment of malignant brain tumors. By replicating biological structures and functions, BNCs enhance drug stability, facilitate penetration across the BBB, and promote tumor-specific targeting while minimizing the risk of systemic toxicity. These nanocarriers can be engineered using a wide variety of biomaterials, including cell membranes, liposomes, and polymeric systems. Recent preclinical and early clinical studies suggest that BNCs provide a viable method to increase drug bioavailability and therapeutic activity. However, several translational challenges remain, including formulation stability, immunogenicity, regulatory acceptability, and large-scale manufacturing. This review discusses recent advances in BNCs for GBM treatment, drug-targeting strategies, and the structural hurdles that limit their clinical translation. Furthermore, we examine emerging multimodal approaches that integrate BNCs with immunotherapy and gene therapy to enhance personalized GBM treatment.

## Simple Summary

Glioblastoma (GBM) is the most aggressive and common brain tumor, highly resistant to conventional treatment, largely due to the blood brain barrier (BBB) limiting effective drug delivery. Biomimetic nanoparticles (BNPs) offer a promising therapeutic approach by mimicking biological structures to improve drug stability, BBB penetration, and tumorspecific targeting while reducing systemic toxicity. BNPs can be engineered from diverse biomaterials, including cell membranes, liposomes, and polymeric systems. Preclinical and early clinical studies show BNPs enhance drug bioavailability and therapeutic efficacy. However, challenges remain stability, immunogenicity, regulatory approval, and scalable production hindering clinical translation. This review highlights recent BNPs advances, targeting strategies, and limitations, while exploring integration with immunotherapy and gene therapy to enable more personalized, effective GBM treatment approaches.

## 1. Introduction

Glioblastoma (GBM) is the most aggressive and lethal type of glioma, accounting for approximately 50% of all glioma cases. Classified by the World Health Organization (WHO) as a Grade IV astrocytoma, GBM is characterized by rapid growth, deep tissue infiltration, and profound resistance to standard clinical care [1]. Despite advancements in radiation, chemotherapy, and surgical resection, the median survival for a GBM patient remains approximately 15 months post-diagnosis. This poor prognosis is primarily driven by extensive tumor genetic heterogeneity, the invasive nature of GBM cells, and the presence of an intact bloodbrain barrier (BBB) that severely restricts therapeutic agents from reaching the brain parenchyma [2].

GBM arises from astrocytic glial cells and exhibits a highly malignant phenotype driven by distinct molecular and histological characteristics. Histologically, it features pseudopalisading cells surrounding hypoxic and necrotic areas, supported by a highly vascularized microenvironment. The pathogenesis of GBM involves multiple genetic mutations and aberrant signaling pathways, including alterations in EGFR, PTEN, and TP53. These molecular aberrations contribute to inherent drug resistance and complicate targeted therapeutic interventions [3].

A critical area of development in GBM treatment is targeted therapy, which is designed to selectively deliver therapeutic agents to tumor cells while mitigating systemic off-target effects. Unlike traditional chemotherapy, which indiscriminately affects both rapidly dividing healthy and cancerous cells, targeted therapies exploit specific molecular markers and signal transduction networks overexpressed in tumor cells to enhance drug specificity and efficacy [4]. Various targeted modalities are currently under investigation for GBM, including gene therapy, small-molecule antagonists, and monoclonal antibodies (e.g., bevacizumab) [5].

**Figure 1:**
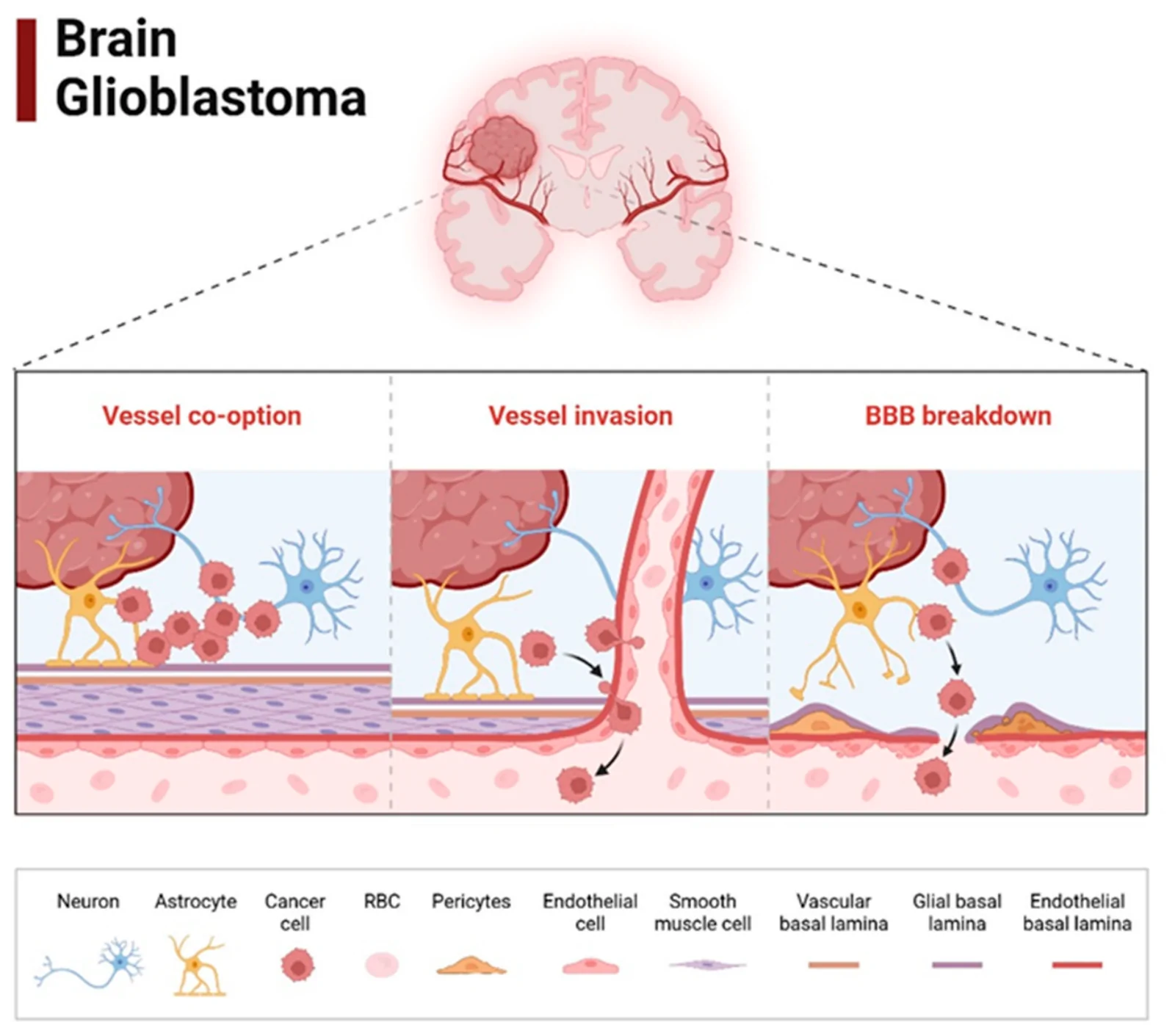
Schematic representation of biomimetic nanocarrier strategies for glioblastoma. The workflow illustrates the critical relationship between specific nanoparticle design (biomaterial coatings), the overcoming of biological barriers (the blood–brain barrier), active targeting mechanisms (ligand-receptor binding), and the resulting therapeutic outcomes (targeted tumor eradication).

By mimicking biological processes, biomimetic nanoparticles improve the effectiveness of therapeutic interventions (Figure 1). Nanoparticles like these are designed to engage with the cancer microenvironment in one of three ways: surface modification [6,7], ligand-receptor interaction, or encapsulation in cell membranes. Polymeric nanoparticles and hybrid biomaterials are two of the many biomimetic nanoparticles being studied for the potential to treat cancer. Optimising BNP use in GBM treatment will also necessitate advancements in bioengineering, next-gen nanomaterials, and AI-based medication design [8].

The innovative strategy aiming to address the treatment constraints of GBM includes biomimetic nanoparticles. Despite certain development and clinical translational hurdles, biomimicry ideas are continually improving the design and further refining therapeutics; this is in addition to biomimetic nanoparticles’ enhanced targeting, penetration across the BBB, and drug release on demand. They hold great potential for improving the treatment of GBM patients soon when combined with current therapy procedures and explored in forthcoming nanotechnology research [9].

Unlike previous reviews that have focused on nanomedicine for neuroinflammation [10], CPPs-PLGA nanocarriers [11], CXCL12-functionalized hydrogels [12], or polymer-based nanomedicines for GBM [13], our review uniquely integrates biomimetic nanocarriers with translational clinical challenges in glioblastoma therapy. The novelty of this work lies in its systematic approach that connects biomimetic design principles with immunotherapy and gene therapy strategies, while also addressing practical issues such as stability, immunogenicity, and large-scale production. In doing so, this review provides a comprehensive translational framework that bridges preclinical research with clinical application, positioning biomimetic nanocarriers as next-generation therapeutic strategies for GBM.

Thus, the ultimate goal of this review is to provide a translational roadmap for biomimetic nanocarriers in glioblastoma therapy, bridging fundamental nanomaterial design with clinical applicability. By synthesizing current advances and identifying strategic challenges, including stability, immunogenicity, regulatory hurdles, and scalable production, this work aims to guide future research toward clinically viable solutions. In doing so, the review seeks to position biomimetic nanocarriers not only as innovative drug delivery systems but also as integral components of multimodal therapeutic strategies that can enhance patient survival and quality of life [3].

In this review, the term “nanocarriers” is used as the overarching term to describe nanoscale delivery systems designed to transport therapeutic agents to glioblastoma tissues. “Nanoparticles” is used more specifically to refer to particulate nanoscale systems, including polymeric nanoparticles, lipid nanoparticles, metallic nanoparticles, and related particulate platforms. Biomimetic nanocarriers may therefore include both nanoparticle-based systems and other nanoscale delivery platforms, such as extracellular-vesicle-inspired systems, cell-membrane-coated nanoparticles, exosome-based carriers, and biomimetic hybrid systems. This distinction is maintained throughout the manuscript to ensure consistent and precise terminology.

Despite the growing number of reviews addressing nanomedicine and nanoparticle-based drug delivery for glioblastoma, an important gap remains in the critical evaluation of biomimetic nanocarriers as biologically inspired therapeutic platforms. Previous reviews have broadly examined nanomaterials for overcoming the blood brain barrier (BBB), improving drug delivery, or enabling targeted therapy; however, the present review specifically focuses on how biomimetic design principles can reproduce or exploit biological structures, interfaces, and endogenous transport mechanisms to address the complex barriers encountered in glioblastoma. The unique contribution of this review is its integrated analysis of biomimetic nanocarriers across BBB and brain tumor barrier transport, cell-specific targeting, immunotherapy, gene therapy, and multifunctional therapeutic strategies, while simultaneously evaluating the biological limitations and translational challenges associated with these approaches. Particular attention is given to the distinction between promising preclinical performance and genuine clinical translation, including issues of tumor heterogeneity, infiltrative disease, biodistribution, immunogenicity, formulation stability, manufacturing reproducibility, scalability, safety, and regulatory requirements. Rather than presenting biomimetic nanocarriers as uniformly superior to conventional nanomedicines, this review critically examines their potential advantages, limitations, and evidence gaps and identifies the conditions under which biomimetic engineering may provide a meaningful therapeutic advantage in GBM. This integrated perspective is intended to provide a clearer framework for assessing the current maturity of biomimetic nanocarriers and for guiding the development of clinically relevant next-generation GBM nanotherapeutics.

## 2. Biomimetic Nanocarriers in Biological Systems

Biomimetic nanocarriers (BNCs) are engineered nanoscale delivery systems designed to pass themselves off as part of a living organism [14]. To ensure clarity throughout this review, we utilize the term biomimetic nanocarrier to describe the fully assembled, biologically inspired delivery vehicle, distinguishing it from the underlying synthetic nanoparticle core. This field is a prime example of biomimicry, which seeks to create materials that imitate biological systems, functions, or processes by utilizing the laws of nature [15]. The field known as “biomimicry of nanomaterials” seeks to improve upon traditional synthetic materials by modeling their properties and functions after those found in nature at the nanoscale [16]. These nanocarriers have a number of potential uses, including medicine delivery, environmental cleanup, and diagnostics, because they aim to imitate behavior, properties, and even natural self-assembly processes [17].

The capacity of natural systems to build complex, highly efficient ones with little input is the source of inspiration for biomimicry. Examples of complex systems that organisms in nature have evolved to carry out essential functions include the formation of cell membranes, the immune system’s reaction to infections, and bacterial creation of nanostructures [18]. They do not need externally supplied energy, save energy and are extremely versatile as living entities. Mimetic nanoparticles try to imitate these systems by means of natural processes and materials. For example, inspired by the architecture of cell membranes, nano-scale particles have been fabricated to enter biological systems without killing them or reacting in a harmful immunogenic manner [19]. Nanoparticles have gained increasing application in targeted drug delivery because they can protect therapeutic agents from metabolic degradation, enhance delivery to specific cells or tissues, and facilitate controlled release [20].

### 2.1. Material-Based Classification of Biomimetic Nanocarriers

To effectively navigate the complex biological barriers of glioblastoma (GBM), biomimetic nanocarriers (BNCs) can be broadly categorized into two main groups based on their fundamental materials and surface modifications: naturally derived biological carriers and surface-modified synthetic nanocarriers.

#### 2.1.1. Natural and Biopolymer-Based Carriers

This category includes nanocarriers that are inherently biological in origin.

Exosomes and Extracellular Vesicles: Secreted by almost all cell types, these lipid-bilayer vesicles naturally facilitate intercellular communication. Because they possess inherent biocompatibility and homing capabilities, exosomes derived from immune cells or even tumor cells can effectively cross the blood–brain barrier (BBB).

Protein and Biopolymer Constructs: Carriers synthesized from natural biopolymers (e.g., albumin, chitosan, or hyaluronic acid) offer exceptional biodegradability and low immunogenicity. Albumin-based carriers, for example, leverage endogenous transport pathways (such as SPARC proteins) to infiltrate tumor tissues.

#### 2.1.2. Surface-Modified Synthetic Carriers

This approach combines the highly tunable physical properties of synthetic nanomaterials with the biological stealth of natural membranes.

Synthetic Polymers: Polymeric cores, such as PLGA or PEGylated systems, are frequently utilized for their high drug-loading capacity and controlled release profiles. By modifying their surfaces with specific targeting ligands or peptides, these synthetic cores can mimic biological behaviors.

Inorganic and Metallic Materials: Mesoporous silica, gold nanoparticles, and iron oxide nanoparticles offer unique theranostic capabilities, including magnetic resonance imaging (MRI) contrast or photothermal therapy.

Cell-Membrane Camouflaging: A premier strategy in this category involves extruding living cell membranes (from erythrocytes, macrophages, or cancer cells) and coating them directly onto synthetic polymeric or inorganic cores. This “top-down” surface modification grants the synthetic core homologous targeting abilities and protection from reticuloendothelial system (RES) clearance.

### 2.2. Cargo Loading and Carrier-Drug Relationships

The design of the BNC is intrinsically linked to the physicochemical properties of the therapeutic cargo it is intended to deliver. The structural relationship between the carrier and the drug dictates the loading efficiency, protection from degradation, and release kinetics within the GBM microenvironment.

**Antitumor Chemicals (Small Molecules):** Hydrophobic chemotherapeutics, such as paclitaxel or doxorubicin, are typically encapsulated within the hydrophobic core of polymeric nanocarriers or the lipid bilayer of liposomes and exosomes. This encapsulation shields the toxic payload from healthy neural tissue, releasing the chemical primarily within the acidic tumor microenvironment.

**Nucleic Acids (siRNA, miRNA, and DNA):** Gene therapy requires protecting fragile nucleic acids from ubiquitous nucleases in the bloodstream. BNCs—particularly cationic lipid-based carriers or exosomes—form stable electrostatic complexes with negatively charged siRNA or mRNA, facilitating safe transit across the BBB and efficient endosomal escape for successful gene silencing or editing.

**Proteins and Peptides:** The delivery of therapeutic proteins, including monoclonal antibodies or immune-stimulating cytokines, relies on carriers that prevent premature enzymatic degradation and denaturation, often utilizing hollow-core biopolymers or carefully engineered liposomal compartments.

### 2.3. Types of Biomimetic Nanocarriers

BNPs consist of several distinct types, each characterized by specific beneficial features and inherent limitations. Among these, nanoparticles such as lipid-, polymer- or protein-based are among the most used. Regarding bio-systems made with phospholipids, lipid particles, including liposomes, are prepared to mimic the structure of the cell membrane [21]. Both hydrophilic drugs, such as doxorubicin (DOX) and some photosensitizers are described, which can be trapped with high efficiency in lipid nanoparticles [22]. Release of the drug is mainly determined by its retention or positional stability not only in the matrix but also within individual lamellae. Polymeric nanoparticles, whether natural or synthetic, have been considered to duplicate the activity of biopolymers produced naturally by living organisms, for example proteins and nucleic acids. One of the applications of nanoparticles is gene delivery, with the aim of improving cell uptake by altering nanoparticle surface properties [23]. Vaccine and enzyme carriers are two beloved application fields for protein nanoparticles, another class of phenomenal biomimetic nanoparticles. Such nanoparticles are created out of native proteins, can form extremely stable conformations spontaneously, and have the advantage of being biocompatible and targeted to specific cells or tissues [24].

In addition to these general types, there are specific target biomimetic nanoparticles such as hybrid nanoparticles, which combine multiple materials to improve performance [25] and silica-based nanoparticles, which mimic the mineralized structure in nature. They may be useful for enhancing utility, stability and biocompatibility in both environmental and therapeutic applications by imitating natural self-assembly processes [26]. They are two advanced nanotechnology platforms for targeted drug delivery against diseases such as glioblastoma, cancer, neurodegenerative disorders, and inflammatory conditions, exemplified by biomimetic nanocarriers and functionalized synthetic nanoparticles. The two systems, while aimed at enhancing therapeutic effectiveness and minimizing systemic toxicity, are substantially different in composition, biological behavior, targeting mechanisms, immunogenicity and translational potential.

#### 2.3.1. Biomimetic Nanocarriers

Biomimetic nanocarriers are engineered nanostructures that mimic natural biological structures, including cell membranes, extracellular vesicles or viruses. Exploiting the natural biological characteristics of live organisms, these systems enable their prolonged circulation, immune evasion ability, accurate delivery to blood brain barriers (BBB) and particular targeting to cells. They comprise the fabrication of membrane-coated nanoparticles through a facile process, in which natural cell membranes isolated from red blood cells, platelets, macrophages, stem cells, leukocytes or even whole cancer cells are coated onto synthetic nanoparticle cores (e.g., PLGA, gold nanoparticles and mesoporous silica). Coating with a natural membrane incorporates native membrane proteins and surface antigens that evade immune detection (labeling), as well as targeting ability toward specific tissues.

The exosome-inspired nanoparticles are specifically designed to resemble the natural extracellular vesicles that play a critical role in intercellular communication. Moreover, gold nanoparticles are internalized by cells through endocytosis and accumulate in the nucleus due to their intrinsic targeting properties, making them advantageous carriers for the delivery of siRNA, miRNA, mRNA, proteins, and chemotherapeutic agents across various biological barriers, including the blood–brain barrier (BBB). Virus-like nanoparticles (VLPs) are macromolecular structures that mimic the structural organization of a viral capsid by not having any accompanying infectious genetic material. They have very efficient cellular entry without pathogenicity, thus making them a great vector for vaccines and targeted gene delivery. Biomimetic nanocarriers provide potential benefits of improved biocompatibility, lowered uptake by macrophages (by phagocytosis), increased blood circulation time and tumor specificity as well as greater penetration across the BBB. But they suffer from disadvantages such as complex manufacturing processes, batch-to-batch variability, hurdles in scalability, high production costs and regulatory challenges.

#### 2.3.2. Functionalized Synthetic Nanoparticles

Functionalized synthetic nanoparticles are man-made nanomaterials that are composed of polymers, lipids, metals, silica, dendrimers or carbon-based materials. They are chemically modified on their surface with targeting ligands, antibodies [4,5], peptides, aptamers, folic acid as well as transferrin and/or polyethylene glycol (PEG) to further improve specificity against diseased tissue. By contrast, synthetic nanoparticles do not have intrinsic biological origins and are fully reliant on surface engineering for targeting and beneficial therapeutic activity, in stark comparison to biomimetic systems. The examples include d PLGA nanoparticles, liposomes, dendrimers, gold nanoparticles, magnetic nanoparticles, solid–lipid nanoparticles and polymeric micelles. Functionalized synthetic nanoparticles offer exquisite control over size, surface charge, drug loading capacity, and release kinetics. They are easier to manufacture, very reproducible, scalable for industrial production and more suitable for regulatory approval. However, they can be quickly eliminated by the reticuloendothelial system (RES), provoke immune responses, have limited BBB penetration and require complex surface engineering to ensure effective targeting.

## 3. Mechanisms of Targeted Therapy Using Biomimetic Nanocarriers

BNP-directed therapy is at the forefront of the latest and most advanced treatment strategies for complex diseases such as cancer. The aim of a targeted agent is to maximize the effect on the pathogenic area while minimizing side effects on normal tissue by using a drug [27]. Due to their biocompatibility, stability and, among other things, the ability to selectively bind to receptors on the cell’s surface, BNPs mimicking biological architectures are very promising for therapeutic purposes. It is known that selective targeting and transfection of BNPs in-situ to receptor-overexpressing cells can be achieved through a variety of mechanisms, including ligand-receptor interaction [28]. The targeting of a highly lethal brain tumour, glioblastoma, by the biomimetic nanoparticle signifies the flexibility of our nanoparticle to overcome barriers such as the BBB, which is a major challenge in the treatment of diseases associated with Central Nervous System (CNS) [29].

### 3.1. Targeting Mechanisms via Ligand-Receptor Interactions

**Figure 2:**
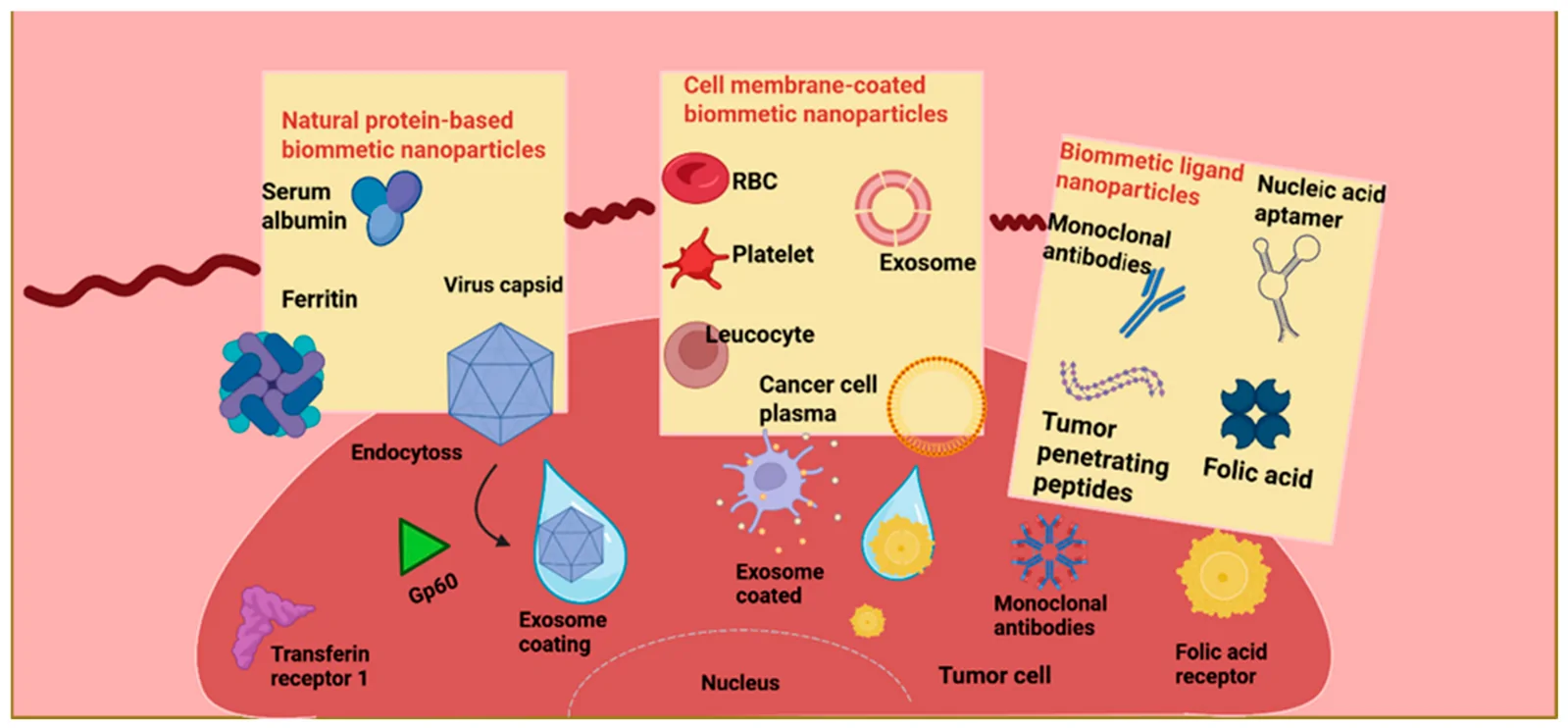
Biomimetic nanoparticles based on various biomaterials with targeted cell entry techniques.

Amongst the most effective BNP-targeted delivery system is that directed at cells with high specificity, i.e., interaction between ligands and receptors [30]. To selectively bind to diseased cells that overexpress surface receptors, the surfaces of biomimetic NPs may be functionalized with peptides, antibodies, or small molecular ligands [31]. These receptors are commonly overexpressed on cancer cells and pathological tissue, which makes them conveniently suitable for targeting purposes (Table 1). For instance, cancer cells frequently overexpress folate receptors; so, the BNPs could be designed in a way that they carry folate molecules and target only cancer cells [32]. In a similar vein, pharmacological targeting has been used to specifically target tumour regions where pro-angiogenic receptors (e.g., integrins or epidermal growth factor receptors (EGFR)) have higher levels than in adjacent healthy tissue [33]. Figure 2 shows the target specificity of biomimetic nanoparticles to different cells.

The ligand-receptor interaction can also be applied to receptor-mediated endocytosis, where the receiving cell uptakes the receptor-nanoparticle complex [34]. The drug payload can be released via enzymatic hydrolysis of the nanoparticle or pH-responsive delivery mechanisms inside the cell to achieve a tumor-targeted and controlled-release effect [35]. Additionally, some surface markers could be specifically targeted by biomimetic nanoparticles for increased treatment selectivity and therapeutic index in minimizing off-target toxicity and thereby increasing the drug effectiveness [36].

**Table 1:** Nanoparticles used for targeted therapy in glioblastoma.

No	Nanoparticle Type	Drug/Agent	Targeting Ligand	Outcome	Reference
1	Solid Lipid NP	Paclitaxel	None	Biodegradable carrier with sustained release	[37]
2	Polymeric NP (PLGA)	Temozolomide	RGD peptide	Targets integrins overexpressed in GBM	[38]
3	Gold NP	Cisplatin	Anti-EGFR antibody	Used for photothermal therapy and drug delivery	[39]
4	Mesoporous Silica NP	Curcumin	Folic acid	Antioxidant and anti-inflammatory properties	[40]
5	Dendrimer	Methotrexate	Lactoferrin	Small size allows BBB penetration	[41]
6	Magnetic NP (SPIONs)	Doxorubicin	Transferrin	MRI-visible, magnetically guided delivery	[42]
7	Carbon Nanotube	Temozolomide	None	High drug loading capacity	[43]
8	Exosome-based NP	siRNA (EGFRvIII)	Natural exosomal proteins	Cell-specific targeting via exosomes	[44]
9	Polymeric Micelle	Docetaxel	cRGD	Enhanced solubility and tumor penetration	[45]
10	Chitosan NP	Etoposide	None	Mucoadhesive and biodegradable	[46]
11	Albumin NP	Paclitaxel (nab-pac)	None	FDA-approved in other cancers; under study in GBM	[47]
12	Graphene Oxide NP	Irinotecan	TAT peptide	Enhanced cellular uptake	[48]
13	PLGA-PEG NP	Carmustine (BCNU)	Angiopep-2	Ligand improves BBB transport	[49]
14	Quantum Dot NP	Fluorouracil (5-FU)	Antibody fragment	Dual imaging and therapy (theranostics)	[50]
15	Polycaprolactone NP	Vincristine	None	Sustained drug release	[51]
16	Iron Oxide NP	siRNA (STAT3)	Aptamer	Gene silencing and imaging capability	[52]
17	Hydrogel NP	TMZ + Resveratrol	None	Injectable gel for localized therapy	[53]
18	Hyaluronic Acid NP	Doxorubicin	CD44	CD44-targeting increases tumor specificity	[54]

### 3.2. Cell-Specific Delivery in Glioblastoma

Because of its aggressiveness, medication resistance, and invasiveness, GBM is a malignant brain tumour that presents complex treatment challenges. The BBB is a selective barrier that hampers the delivery of many therapeutic medications to the site of a GBM tumour, making treatment of the disease extremely difficult [55]. Their surface functionalisation with ligands that enable transport via the BBB’s endothelial cells is responsible for BBB penetration [56]. One method to functionalise BNPs for brain transport is to connect them to transferrin receptors, which are abundant in the BBB and can be found in transferrin-bound ligands like transferrin [57].

The ability of biomimetic nanocarriers to cross the blood–brain barrier (BBB) depends strongly on the interaction between the nanoparticle surface and transport or recognition systems expressed by brain endothelial cells. Among the most frequently investigated strategies, transferrin receptor (TfR)-mediated targeting exploits the natural iron-transport pathway of the BBB. Transferrin or transferrin-mimetic ligands displayed on nanoparticle surfaces can interact with TfR expressed on brain endothelial cells and promote receptor-mediated endocytosis followed by transcytosis. This strategy provides a biologically relevant route for enhancing nanoparticle transport across the BBB compared with nonspecific diffusion. However, TfR is also expressed in peripheral tissues and endogenous transferrin competes for receptor binding. Excessively strong receptor binding may additionally favor endothelial retention and lysosomal trafficking rather than productive transcytosis. Therefore, ligand density, binding affinity, nanoparticle size, and surface architecture are important determinants of whether TfR targeting results in effective BBB transport.

Folate receptor-mediated targeting provides another ligand-based approach, although its relevance to BBB transport and GBM targeting depends on the specific receptor subtype and its expression pattern. Folic acid can be conjugated to nanoparticle surfaces to promote recognition by folate-binding receptors and enhance receptor-mediated cellular uptake. In GBM, this strategy may provide additional tumor-cell recognition when the relevant folate receptor is sufficiently expressed. Its major advantage is the relatively simple and stable chemical conjugation of folic acid to nanocarriers and the potential for improved cellular internalization. Nevertheless, receptor expression is heterogeneous and may differ between tumor cells, brain endothelial cells, and normal tissues. Consequently, folate functionalization should not be interpreted as a universal BBB-crossing mechanism, and its effectiveness is likely to depend on receptor availability and the anatomical distribution of the target.

Epidermal growth factor receptor (EGFR) targeting is particularly relevant to GBM because aberrant EGFR signaling is frequently associated with tumor growth, proliferation, survival, and therapeutic resistance. EGFR-targeting ligands, peptides, or antibodies incorporated into nanoparticles can increase recognition and internalization by EGFR-expressing glioblastoma cells. This approach may therefore provide a second level of specificity after nanoparticle access to the brain or tumor compartment. However, EGFR targeting primarily represents a tumor-cell recognition and internalization strategy rather than an independent mechanism for crossing an intact BBB. Moreover, EGFR expression is heterogeneous across GBM cells, and receptor alterations such as EGFR amplification or mutation do not necessarily result in uniform surface availability. These factors can limit the ability of EGFR-targeted nanocarriers to reach all malignant cell populations, particularly infiltrative cells located outside highly vascularized tumor regions.

Angiopep-2 represents another important targeting ligand because it can interact with low-density lipoprotein receptor-related protein 1 (LRP1), a receptor involved in transport processes at the BBB. LRP1-mediated uptake can facilitate receptor-mediated transcytosis and has therefore been investigated as a strategy for enhancing delivery of therapeutic cargos into the brain. Angiopep-2 functionalization may provide an additional advantage by combining BBB transport with enhanced uptake by certain tumor cells. However, LRP1 expression and trafficking behavior are not uniform across the BBB and tumor compartments, and receptor-mediated transport can be influenced by ligand density, nanoparticle characteristics, and intracellular trafficking pathways. Thus, increased endothelial uptake should be distinguished from productive transcytosis and subsequent delivery of an active therapeutic payload to GBM cells.

Collectively, these targeting strategies illustrate that BBB penetration and tumor targeting are related but distinct processes. Transferrin and Angiopep-2 primarily exploit endogenous receptor-mediated transport pathways to facilitate interaction with brain endothelial cells, whereas EGFR and folate-based approaches can provide greater emphasis on tumor-cell recognition and internalization. A major limitation is that no single receptor is uniformly expressed across the BBB, tumor vasculature, and heterogeneous GBM cell populations. Furthermore, successful receptor binding does not necessarily result in transcytosis, tumor penetration, or therapeutic activity. Biomimetic nanocarriers may therefore benefit from combining complementary targeting mechanisms, for example, a BBB-transport ligand with a tumor-cell-specific ligand, while carefully controlling ligand density and nanoparticle architecture.

Importantly, BBB penetration should not be evaluated solely by measuring nanoparticle accumulation in brain tissue. A meaningful assessment should consider the fraction of nanoparticles that successfully cross the endothelial barrier, their distribution within tumor and non-tumor brain regions, penetration into infiltrative GBM cells, intracellular uptake, payload release, and subsequent therapeutic response. This distinction is particularly important because the BBB is spatially heterogeneous in GBM, with regions of disrupted vascular integrity existing alongside areas in which the barrier remains relatively intact. Consequently, biomimetic targeting strategies should be viewed as complementary mechanisms for overcoming specific biological barriers rather than as universally effective solutions for GBM drug delivery.

Biomimetic nanoparticles have more than just ligand-targeting capabilities; they can also mimic tumours’ increased permeability and retention (EPR) function. Nanoparticles are able to easily enter tumour tissue as contrasted to normal tissue since tumours are characterised by leaky blood vessels [58].

The enhanced permeability and retention (EPR) effect represents a passive mechanism of nanoparticle accumulation that arises from abnormal tumor vasculature and impaired lymphatic drainage. However, the classical EPR effect should not be considered synonymous with biomimicry. Biomimetic nanoparticles are designed to reproduce or exploit biological structures, interfaces, and functions, such as cell-membrane characteristics, endogenous protein interactions, immune evasion, and receptor-mediated transport. Thus, rather than describing biomimetic nanoparticles as “mimicking” the EPR effect, it is more appropriate to consider whether biomimetic surface properties can facilitate interaction with the abnormal tumor vasculature or enhance retention following tumor access.

This distinction is particularly important in glioblastoma, where passive EPR-mediated accumulation is substantially complicated by the blood–brain barrier (BBB) and the heterogeneous blood–brain tumor barrier. Although some GBM regions exhibit abnormal vascular permeability, these characteristics are not uniformly present throughout the tumor, and infiltrative tumor cells may remain in relatively intact brain tissue. Consequently, reliance on the classical EPR effect alone is unlikely to provide sufficiently selective or homogeneous delivery throughout GBM. Biomimetic approaches may therefore provide complementary advantages by incorporating biological recognition mechanisms, including receptor–ligand interactions, cell-membrane camouflage, endogenous protein-mediated transport, and receptor-mediated transcytosis across brain endothelial cells.

Accordingly, the potential advantage of biomimetic nanoparticles in GBM should be discussed primarily in terms of their ability to reproduce biological interfaces and exploit endogenous transport pathways rather than their ability to mimic EPR. Cell-derived membranes, exosome-inspired systems, and biologically functionalized nanoparticles can provide surface characteristics that promote circulation, cellular recognition, BBB interaction, and tumor-cell uptake. These mechanisms may complement passive accumulation where vascular permeability is increased, but they should not be interpreted as evidence that the EPR effect is uniformly operative across GBM.

Therefore, EPR may be regarded as a context-dependent passive delivery phenomenon that can contribute to nanoparticle accumulation in selected tumor regions, whereas biomimetic engineering represents an active design strategy for overcoming biological barriers and improving targeting. This distinction is essential for accurately defining the role of biomimetic nanoparticles in GBM and for avoiding overstatement of passive tumor accumulation as a biomimetic mechanism [59]. To further increase their capacity to release the medication once they have penetrated the BBB and reached the tumour location, newer generations of BNPs can be engineered with stimulus-sensitive features, such as a pH-sensitive or thermo-sensitive coating [60].

Therapeutic strategies for glioblastoma include concurrent or sequential use of chemotherapeutic drugs (e.g., temozolomide), biomimetic nanoparticles and gene therapy, for example, RNA interference molecules. The nanoparticles can be guided to glioblastoma through ligand targeting. Once they arrive, the payload can be distributed and have a “spot” therapeutic effect at the site, with an improved outcome for the patient [56]. In addition, BNPs can be combined with other modalities of therapy such as radiation or immunotherapy to maximize the overall therapeutic efficacy of glioblastoma.

### 3.3. Advances in Biomimetic Nanocarriers for Glioblastoma Therapy

Because of its invasiveness and virulence, as well as the complexity of the BBB, which limits drug access to tumour brain tissue, GBM is among the most challenging malignant diseases to cure [61]. Owing to the challenges of drug delivery to brain tumour tissue, conventional radiation and chemotherapeutic strategies have been suboptimal for the treatment of GBM. However, these issues have new possible solutions due to the discovery of BNPs [62]. For targeted therapy of cancer, especially for GBM, biomimetic nanoparticles (NPs) designed to mimic biological functions or natural structures have been demonstrated to be the most efficacious. Novel nanoparticle architectures, with promising reported positive cases from the clinic and laboratory, as well as the potential to further optimize the nanoparticles’ multifaceted nature through an even more comprehensive treatment regimen, stand out among the most encouraging developments in this field [63].

Increased desire for effective delivery methods that can circumvent the hurdle provided by the BBB has led to tremendous development in biomimetic nanoparticle engineering for glioblastoma therapy in the last couple of years [64]. Nanoparticles functionalised with ligands carrying specific receptors directed at targeting BBB endothelial cells or even glioblastoma cells have proven to be effective. Research has shown that ligand-coated nanoparticles, such as transferrin, folic acid, or EGF receptors, can improve the selectivity of drug delivery to glioblastoma cells [65]. To improve medication administration and decrease off-target adverse effects, ligands enable the nanoparticles to attach to receptors in the cancer cell membrane or the endothelial cells lining the blood–brain barrier [66].

Another area of great interest is the development of nanoparticles with the ability to cross the blood–brain barrier. Due to its selectivity, the blood–brain barrier prevents traditional drug delivery devices from transporting medicinal compounds to the brain [67]. The tiny, surface-modified biomimetic nanoparticles, on the other hand, hold tremendous promise for overcoming this obstacle. Successful drug delivery into glioblastoma cells has been achieved by means of transferrin- or insulin-ligand-functionalised nanoparticles that have been linked to the BBB via a receptor [68]. Some have also been able to briefly pass through the BBB’s tight junctions with the help of protein agonists or targeted ultrasonography, among other treatments, so that the drug’s payload can be delivered.

Nanoparticles have the potential to target chemotherapeutic medications as well as gene editing treatments based on CRISPEN technology or RNA interference (RNAi). Chemotherapy kills cancer cells directly, although both methods aim to target specific genes associated with glioblastoma progression and resistance to treatment. Researchers in [69] developed multifunctional nanoparticles to deliver paclitaxel and siRNA to the drug resistance gene simultaneously. The results showed that glioblastoma cells and animal models benefited substantially from chemotherapy and gene knockdown co-delivery.

Glioblastoma patients may also benefit from imaging probes that use multifunctional BNPs to monitor the administration of drugs in real time. They can follow particles in vivo in real-time with the use of nanoparticles, MRI contrast agents, fluorescent tags, and PET tracers. As a result, doctors can track how therapeutic medications are entering and leaving the tumour, which leads to more targeted treatment plans with fewer unwanted side effects. Researchers used multitasking BNPs loaded with chemotherapeutic drugs and MRI contrast agents to track glioma cell ingress via the blood–brain barrier [70]. To develop treatment regimens based on the delivery’s efficacy, which can be tracked in real-time in vivo, monitoring medicine administration is highly relevant from a therapeutic standpoint (Table 2).

By combining with photothermal or photodynamic therapy, nanoparticles can be tailored to have a synergistic therapeutic effect [71]. These were only released this year in a co-delivery system (doxorubicin as a chemotherapeutic drug and biomimetic particles as photosensitiser) in the trial. In combination with chemotherapy, the ROS generated from the laser-excited photosensitizer would selectively damage the cancer cells to enhance therapy efficiency. As it targets multiple therapeutic strategies with the intention of circumnavigating drug-induced interactions with resistance patterns commonly seen in GBM, this broad-based approach holds great potential for advances in glioblastoma therapy [72].

**Figure 3:**
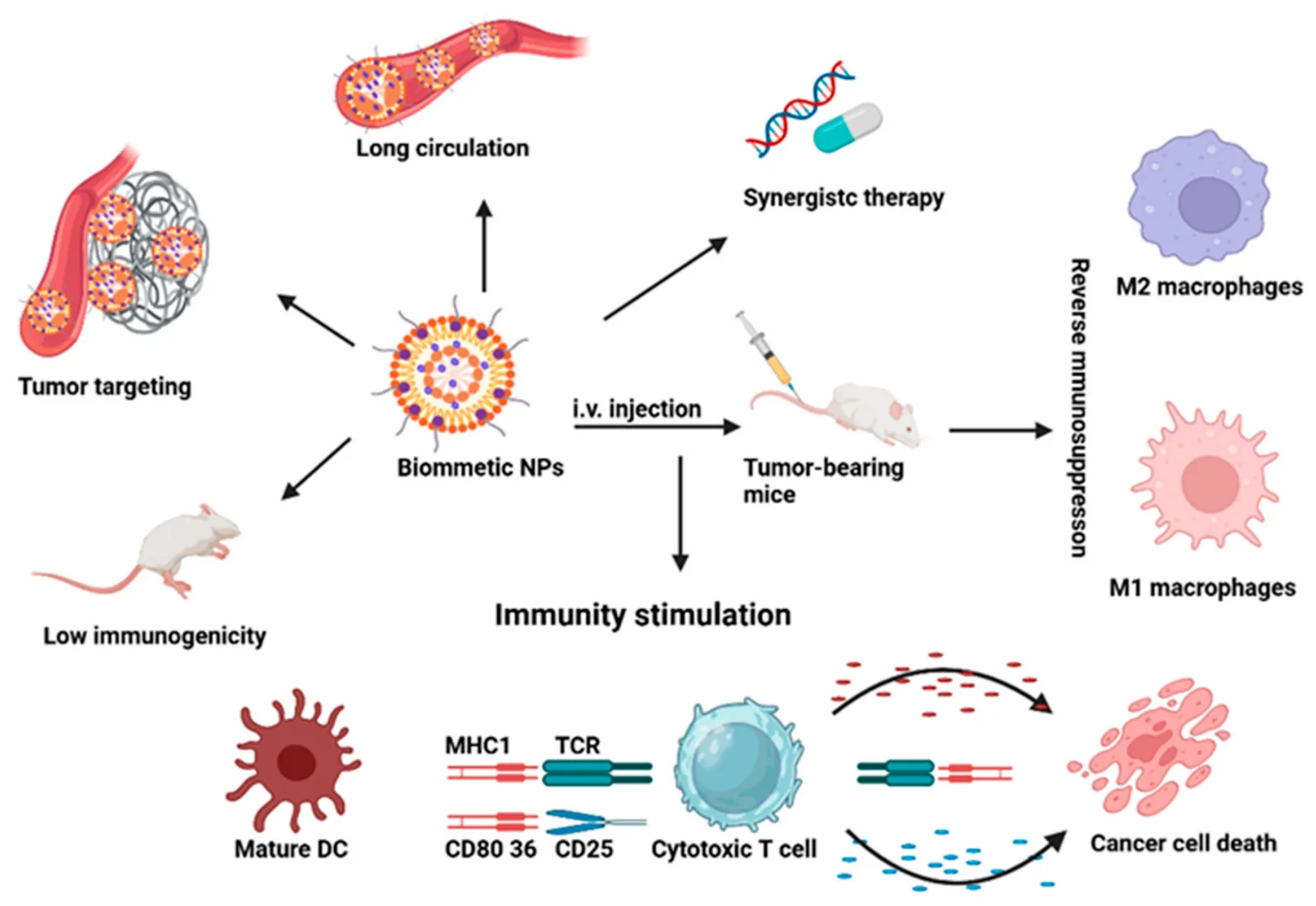
Mechanistic overview connecting biomimetic nanoparticle design with therapeutic outcomes in glioblastoma. Strategic biomaterial selection provides low immunogenicity and prolonged circulation (design), facilitating targeted delivery mechanisms that overcome biological barriers to achieve synergistic therapy and immune stimulation (outcomes). The bidirectional curved arrow indicates a dynamic immune response—T cells recognize tumor antigens (via MHC–TCR interaction), release cytotoxic factors, and induce cancer cell death. Red dots symbolize cytokines or cytotoxic molecules (like perforin and granzyme) released by activated T cells. Blue dots represent antigens or signaling molecules exchanged between cancer cells and immune cells.

BNCs offer a highly targeted and strategic drug delivery platform that demonstrates significant potential against glioblastoma in preclinical models. Multimodal nanocarrier systems endow glioblastoma therapies with greater selectivity by leveraging novel surface architectures that facilitate specific ligand-receptor binding and environmental responsiveness (Figure 3). BNCs have shown promise in in vivo studies by successfully crossing the BBB and actively targeting glioma cells, which can enhance therapeutic efficacy while reducing systemic toxicity. Due to their multi-functionality, these nanocarriers facilitate complex treatment regimens by allowing the simultaneous co-delivery of imaging agents, gene therapies, and chemotherapeutic drugs. Continued optimization of biomimetic nanocarriers holds the potential to significantly advance glioblastoma treatment strategies and improve patient outcomes [73].

**Table 2:** Recent innovations and developments in biomimetic nanoparticle engineering for glioblastoma therapy.

No	Innovation Type	Biomimetic Component	Core Nanoparticle	Targeting Mechanism	Inference	Reference
1	Cell membrane-coated NP	Red Blood Cell (RBC) membrane	PLGA	Prolonged circulation	Reduces immune clearance	[74]
2	Leukocyte-mimetic NP	Monocyte membrane	Liposome	Inflammation-targeted	Mimics immune cell trafficking	[75]
3	Cancer cell membrane-coated NP	Glioma cell membrane	Gold NP	Homotypic targeting	Enhances tumor uptake	[76]
4	Hybrid membrane NP	RBC + glioma cell membrane	Mesoporous silica NP	Dual targeting	Combines long circulation with tumor affinity	[77]
5	Exosome-based NP	Natural exosomes	siRNA-loaded vesicles	Intrinsic homing	Ideal for gene delivery	[78]
6	Platelet-mimetic NP	Platelet membrane	PLGA	Tumor vasculature targeting	Useful for targeting glioma vasculature	[79]
7	Stem cell-mimetic NP	Mesenchymal stem cell membrane	Lipid-polymer hybrid NP	Tumor tropism	Migrates toward glioblastoma niches	[80]
8	Neutrophil-like NP	Neutrophil membrane	Magnetic NP	Inflammatory targeting	For BBB crossing in tumor inflammation	[81]
9	Bacterial vesicle-inspired NP	Outer membrane vesicles	Polymeric NP	Pathogen-associated targeting	Activates immune response too	[82]
10	T-cell membrane-coated NP	Cytotoxic T-cell membrane	PLGA	Immune recognition	Dual function: therapy + immune activation	[83]
11	Macrophage-mimetic NP	M1 macrophage membrane	PLGA-PEG	Infiltrates tumor microenvironment	Supports immunotherapy strategies	[84]
12	Virus-like NP	Viral capsid mimicry	Protein-based NP	Natural BBB crossing	Engineered viral vectors without pathogenicity	[85]
13	Dual-ligand biomimetic NP	Folic acid + membrane coating	Gold NP	Enhanced endocytosis	Multifunctional design	[86]
14	Glucose-modified membrane NP	RBC membrane + glucose ligand	PLGA	GLUT1 targeting on BBB	Facilitates BBB transcytosis	[87]
15	Antibody-decorated biomimetic NP	Exosome + anti-EGFR antibody	Liposome	EGFR targeting	Precision delivery to EGFR + GBM cells	[88]
16	ApoE-functionalized membrane NP	Brain-targeting peptide	Solid lipid NP	LDL receptor-mediated transport	BBB transport via receptor targeting	[89]
17	Artificial exosome NP	Synthetic lipid bilayer	siRNA/polymer complex	Biomimic exosomes for RNA delivery	Tunable and customizable cargo	[90]
18	Peptide-camouflaged NP	Angiopep-2 peptide + membrane	Mesoporous silica NP	LRP1-mediated BBB transport	Widely used for brain targeting	[91]
19	Biomimetic nanogel	Cell membrane-embedded gel	Hydrogel	Sustained release in tumor niche	Injectable for local GBM therapy	[92]
20	CRISPR-loaded biomimetic NP	Exosome membrane	Lipid-polymer hybrid	Gene editing inside tumor	For GBM gene therapy innovations	[64]

### 3.4. Comparative Evaluation of Biomimetic Nanocarrier Platforms for Glioblastoma Therapy

Although biomimetic nanocarriers share the common objective of improving tumor-specific delivery, their therapeutic performance is strongly influenced by their composition, biological source, surface characteristics, cargo compatibility, and manufacturing method. Therefore, the different biomimetic platforms should not be considered interchangeable. A critical comparison is required because a nanocarrier that demonstrates efficient BBB transport may not necessarily provide optimal drug loading, therapeutic efficacy, safety, or clinical scalability.

Cell-membrane-coated nanoparticles provide an important advantage through the retention of selected biological surface characteristics, which can facilitate immune evasion, prolonged circulation, and interactions with specific receptors. Their synthetic nanoparticle cores can accommodate different therapeutic cargos and provide greater control over particle size and drug release than some naturally derived systems. However, membrane isolation, purification, membrane orientation, and batch-to-batch consistency remain important challenges. Variability in the biological source of the membrane can also influence physicochemical properties and biological performance, thereby complicating standardized manufacturing and regulatory characterization.

Exosome-based and other extracellular-vesicle-derived carriers provide intrinsic biological characteristics that may facilitate cellular uptake and intercellular communication. Their endogenous membrane composition can support efficient delivery of nucleic acids, proteins, and other biologically active cargos. Nevertheless, relatively limited cargo-loading capacity for some therapeutic agents, heterogeneous vesicle populations, source-dependent variability, difficulties in large-scale isolation and purification, and uncertainty regarding long-term safety can restrict their translation. Thus, although exosome-based systems are attractive for highly specific biological delivery, their manufacturing complexity may be greater than that of fully synthetic or hybrid platforms.

Liposomal and lipid-based nanocarriers offer comparatively well-established formulation technologies and can accommodate both hydrophilic and hydrophobic therapeutic agents. Their surfaces can also be modified with targeting ligands to improve tumor accumulation and receptor-mediated uptake. However, lipid-based systems may exhibit limited structural stability, premature drug leakage, rapid clearance, or insufficient penetration into heterogeneous tumor regions unless appropriately engineered. Their relatively mature manufacturing infrastructure provides an important translational advantage, but additional functionalization required for BBB and GBM targeting may increase formulation complexity.

Polymeric and polymer–lipid hybrid nanoparticles provide substantial flexibility in drug encapsulation, controlled release, and surface functionalization. Their physicochemical properties can be systematically modified to regulate particle size, surface charge, degradation, and ligand presentation. These characteristics may provide advantages for combination therapy and co-delivery of chemotherapeutic, gene-regulating, or immunomodulatory agents. However, increasing formulation complexity may also increase manufacturing burden, characterization requirements, and regulatory challenges. In addition, the relationship between particle physicochemical properties and biological behavior must be carefully established before clinical translation.

Peptide-functionalized and ligand-mediated biomimetic systems are particularly relevant to GBM because receptors such as EGFR, transferrin receptor, and LRP1 can provide opportunities for receptor-mediated transport and tumor targeting. These systems may improve cellular specificity and facilitate BBB transport when the selected ligand interacts effectively with the relevant receptor. Nevertheless, receptor expression is heterogeneous between patients and even between different regions of the same tumor. Consequently, high targeting efficiency in receptor-enriched experimental models may not be directly reproduced in patients with heterogeneous GBM.

Biomimetic nanogels and hydrogel-associated systems provide additional opportunities for localized and sustained drug release within the tumor microenvironment. Their ability to retain therapeutic agents may reduce the need for repeated systemic administration and potentially improve local drug exposure. However, their applicability may be restricted by administration route, tissue distribution, mechanical properties, degradation behavior, and difficulties in achieving reproducible large-scale production.

Overall, no single biomimetic nanocarrier platform simultaneously provides optimal drug-loading capacity, BBB penetration, tumor specificity, therapeutic efficacy, safety, stability, and manufacturing scalability. Rather, each platform involves a trade-off between biological functionality and pharmaceutical controllability. Naturally derived systems such as exosomes may offer sophisticated biological interactions but can present greater heterogeneity and manufacturing challenges, whereas synthetic and hybrid systems generally provide better control over formulation parameters but may require additional biomimetic modification to achieve comparable biological targeting.

Importantly, improved BBB transport should not be interpreted as a direct surrogate for clinical efficacy. BBB penetration represents only one component of successful GBM drug delivery. After reaching the brain, nanoparticles must distribute within a highly heterogeneous tumor, interact with the tumor microenvironment, release their cargo at the appropriate site, and maintain sufficient therapeutic exposure while minimizing toxicity. Furthermore, differences in BBB integrity, tumor vascularity, receptor expression, immune status, and extracellular matrix composition among patients can substantially influence therapeutic performance.

From a translational perspective, manufacturing reproducibility represents one of the major limitations of biomimetic nanocarriers. The present manuscript already highlights that variations in particle size, zeta potential, drug loading, and surface characteristics can occur between batches and may complicate scale-up and therapeutic consistency. Protein-corona formation represents an additional concern because adsorption of proteins and other biomolecules can modify the biological identity of nanoparticles and potentially alter targeting efficiency, cellular uptake, and biodistribution. Therefore, future development should prioritize standardized manufacturing, rigorous physicochemical characterization, reproducible biological activity, long-term safety assessment, and clinically relevant GBM models.

A useful translational framework should therefore evaluate biomimetic nanocarriers across multiple dimensions rather than relying solely on tumor growth inhibition or BBB penetration. Systems demonstrating a balanced combination of drug-loading efficiency, controlled release, BBB transport, tumor specificity, therapeutic benefit, safety, manufacturing reproducibility, and regulatory feasibility are more likely to progress successfully toward clinical application.

### 3.5. Critical Analysis of Biomimetic Nanocarriers for Glioblastoma

Although biomimetic nanocarriers have demonstrated considerable potential for glioblastoma therapy, the reported benefits should be interpreted in the context of the biological and technological limitations of the experimental models used. A major advantage of biomimetic systems is their ability to exploit endogenous biological interfaces, such as cell-membrane characteristics, receptor–ligand interactions, and endogenous transport pathways, which may improve circulation, BBB interaction, immune evasion, or tumor-cell recognition. However, enhanced targeting or cellular uptake does not necessarily translate into improved therapeutic efficacy. Many studies demonstrating increased nanoparticle accumulation or uptake have been performed in simplified in vitro systems or animal models that cannot fully reproduce the heterogeneous BBB/brain–tumor barrier, infiltrative growth pattern, extracellular matrix, vascular architecture, and immunological environment of human GBM. Therefore, increased accumulation should be distinguished from effective penetration throughout the tumor and from clinically meaningful therapeutic benefit.

The different biomimetic strategies also involve important trade-offs. Cell-membrane-coated nanoparticles can provide biological recognition and immune-evasive properties, but their performance may depend on membrane source, membrane integrity, coating uniformity, and reproducibility during large-scale manufacturing. Exosome-inspired or extracellular-vesicle-based systems may provide efficient biological communication and cargo delivery, but their heterogeneity, purification, loading efficiency, storage stability, and production scalability remain significant challenges. Ligand-functionalized nanoparticles can improve receptor-mediated targeting, but receptor expression is heterogeneous among GBM cells and may change during tumor progression, potentially limiting the uniformity of targeting. Similarly, multifunctional nanoparticles that combine BBB penetration, tumor targeting, controlled drug release, imaging, immunomodulation, or gene delivery may provide several therapeutic functions simultaneously, but increasing formulation complexity can also increase manufacturing difficulties, batch-to-batch variability, toxicity risks, and regulatory barriers.

Another important consideration is that the apparent superiority of biomimetic nanoparticles is highly dependent on the comparator and experimental endpoint. Improvements in drug loading, cellular uptake, or tumor accumulation do not necessarily demonstrate superiority over conventional nanoparticles or clinically established delivery approaches. Comparative studies using equivalent drug doses, standardized pharmacokinetic measurements, biodistribution analysis, therapeutic endpoints, and toxicity assessments are therefore needed to determine whether biomimetic engineering provides a meaningful therapeutic advantage. In addition, the use of different GBM models, nanoparticle compositions, administration routes, and experimental endpoints makes direct comparison among published studies difficult. Greater standardization would improve the reliability and translational relevance of future investigations.

Importantly, the majority of evidence supporting biomimetic nanocarriers for GBM remains preclinical. Promising results from cellular and animal models provide important proof of concept but should not be considered equivalent to clinical efficacy. Translation to patients requires demonstration of reproducible BBB/brain–tumor barrier transport, adequate penetration into infiltrative tumor regions, predictable pharmacokinetics, long-term safety, low immunogenicity, scalable manufacturing, and consistent product quality. Thus, the current evidence supports biomimetic nanocarriers as a promising experimental platform rather than an established clinical solution. Future studies should prioritize clinically relevant GBM models, rigorous head-to-head comparisons with conventional nanocarriers, longitudinal biodistribution and toxicity assessment, and early consideration of manufacturing and regulatory requirements. Such approaches will help determine whether the biological advantages of biomimetic design can ultimately be converted into a meaningful therapeutic benefit for patients with GBM.

## 4. Successful Case Studies and Clinical Trials

Preclinical and early-clinical studies represent different stages of the translational development of biomimetic nanoparticles and should therefore be considered separately. Preclinical evidence is generated through in vitro experiments, cellular models, organoids, ex vivo systems, and animal models and is primarily used to evaluate nanoparticle physicochemical properties, biological interactions, blood–brain barrier (BBB) transport, biodistribution, pharmacokinetics, therapeutic efficacy, and preliminary safety [93]. In glioblastoma (GBM), most investigations of biomimetic nanoparticles remain at the preclinical stage. These studies have demonstrated that biologically derived membranes, endogenous proteins, extracellular vesicle-inspired systems, and other biomimetic interfaces can potentially improve circulation, facilitate BBB interaction, promote tumor-cell recognition, and enhance the delivery of therapeutic agents. However, findings obtained from animal or cellular models should not be interpreted as evidence of clinical efficacy in patients [94].

Early-clinical evidence, in contrast, refers specifically to investigations conducted in human participants. Early-phase clinical studies, particularly Phase I and Phase I/II trials, primarily assess safety, tolerability, pharmacokinetics, feasibility, and preliminary therapeutic activity rather than definitive efficacy [95]. Importantly, evidence that a nanoparticle formulation is effective in a GBM animal model does not establish that the same formulation has demonstrated clinical benefit in patients. Differences in tumor heterogeneity, BBB/BTB integrity, nanoparticle distribution, immune responses, pharmacokinetics, and tumor microenvironment between experimental models and patients can substantially influence therapeutic outcomes [96].

Accordingly, the current evidence for biomimetic nanoparticle-based GBM therapy should be interpreted predominantly within a preclinical framework. Although some nanomedicine platforms and biomimetic-inspired delivery strategies have progressed toward human investigation, clinical translation of biomimetic nanoparticles specifically for GBM remains limited [95]. Early-clinical studies, where available, should therefore be discussed separately from preclinical studies and should not be used to generalize efficacy demonstrated in animal models. Clear separation of these evidence levels is essential for accurately assessing the translational maturity of biomimetic nanoparticle systems [97].

The transition from preclinical validation to early clinical investigation requires additional evaluation of long-term toxicity, immunogenicity, manufacturing reproducibility, batch-to-batch consistency, scalability, pharmacokinetic behavior, and regulatory requirements. For GBM, further challenges include achieving reproducible transport across the BBB/BTB, penetrating infiltrative tumor regions, maintaining therapeutic concentrations within heterogeneous tumor tissue, and minimizing off-target accumulation. Thus, successful preclinical performance should be regarded as an important foundation for clinical translation, but not as a substitute for human clinical evidence.

The translational development of biomimetic nanocarriers for glioblastoma should be considered according to distinct levels of evidence, ranging from in vitro investigations and animal studies to early clinical evaluation and clinically validated nanomedicine platforms. These stages should not be considered equivalent because each provides different information regarding biological activity, safety, pharmacokinetics, therapeutic efficacy, and clinical feasibility. At present, the majority of biomimetic nanocarriers specifically developed for GBM remain at the preclinical stage. In vitro studies provide initial evidence regarding cellular uptake, receptor recognition, cytotoxicity, drug release, and molecular mechanisms, whereas animal studies provide additional information regarding biodistribution, BBB penetration, tumor accumulation, pharmacokinetics, therapeutic efficacy, and preliminary toxicity. Although these findings are essential for establishing proof of concept, they do not demonstrate clinical efficacy in patients.

Animal models are particularly useful for evaluating whether biomimetic properties translate into improved delivery across the BBB and into GBM tissue. However, their predictive value is limited by differences between experimental models and human disease. Rodent GBM models may not fully reproduce the molecular heterogeneity, infiltrative growth, vascular characteristics, immune microenvironment, and heterogeneous BBB/brain–tumor barrier observed in patients. Consequently, increased nanoparticle accumulation, prolonged survival, or improved tumor regression in an animal model should be interpreted as preclinical evidence rather than as an indication of established clinical effectiveness. Greater emphasis should therefore be placed on clinically relevant models, reproducible pharmacokinetic and biodistribution measurements, and comparative evaluation against appropriate conventional delivery systems.

Early clinical investigations represent a separate stage of development because they involve human participants. Phase I and early Phase I/II studies generally focus on safety, tolerability, pharmacokinetics, feasibility, dose selection, and preliminary evidence of therapeutic activity. These studies should not be interpreted in the same manner as preclinical experiments, and early evidence of safety or feasibility does not establish therapeutic efficacy. Importantly, the availability of an early clinical investigation for a nanoparticle platform does not necessarily indicate that the biomimetic formulation has demonstrated clinical benefit in GBM. Clinical claims should therefore be restricted to evidence obtained directly from human studies and should be clearly distinguished from findings derived from cellular or animal models.

Clinically validated nanomedicine platforms provide an additional but distinct reference point for evaluating the translational potential of biomimetic systems. Several nanoparticle-based technologies have achieved clinical validation for specific therapeutic applications, demonstrating that nanoscale drug delivery can overcome manufacturing, safety, pharmacokinetic, and regulatory barriers. However, clinical validation of a conventional nanomedicine does not establish the clinical effectiveness of a biomimetic nanocarrier, nor does it demonstrate that the same platform will be effective against GBM. Biomimetic systems often involve additional biological components, such as cell membranes, extracellular vesicles, proteins, or complex surface architectures, which can introduce additional challenges related to characterization, stability, immunogenicity, batch-to-batch consistency, and scalable manufacturing.

Therefore, the current evidence should be interpreted cautiously. Biomimetic nanocarriers have generated encouraging preclinical findings by providing potential advantages in BBB interaction, biological recognition, tumor targeting, immune modulation, and therapeutic cargo delivery. Nevertheless, these advantages have not yet been sufficiently established as clinically meaningful improvements for patients with GBM. A successful transition toward clinical application will require systematic validation of BBB/brain–tumor barrier transport, penetration into infiltrative tumor regions, biodistribution, pharmacokinetics, long-term toxicity, immunogenicity, manufacturing reproducibility, scalability, and regulatory compliance. Distinguishing these evidence levels is essential to avoid conflating experimental promise with clinical maturity and to provide a realistic assessment of the translational status of biomimetic nanocarriers for GBM.

## 5. Challenges in the Development of Biomimetic Nanoparticles

Interdependent manipulation of size, surface properties, composition and functionalization is indispensable in the fabrication process for achieving the best results in biomimetic nanoparticle formation. One of the major obstacles that limits BNP synthesis is ensuring that the BNPs are consistent from batch to batch. The size, zeta potential and drug loading of nanoparticles differ significantly from batch to batch due to the intrinsically random production process. These differences are likely to make it more difficult for BNPs to scale up for mass production and lower their therapeutic potency [14]. Standardized manufacturing processes are necessary to overcome this problem and ensure consistent therapeutic use and batch-to-batch uniformity of BNPs.

Significant interpatient pharmacokinetic (PK) variability is one of the most important translational challenges facing biomimetic nanocarriers and functionalized synthetic nanoparticles. There are various factors that determine the biodistribution of nanoparticles, such as particle size, shape, surface charge and composition, ligand density (n), route of administration and physiological state of the patient. Nanoparticle absorption, distribution, metabolism and excretion (ADME) are highly impacted by individual characteristics such as age, body mass index, liver and kidney function, inflammatory status, tumor burden and blood–brain barrier integrity. Differences in nanoparticle accumulation in tumor tissues are due to the heterogeneous nature of tumor vasculature and an incomparably variable blood–brain barrier disruption as seen in glioblastoma.

Nanoparticles immediately adsorb proteins, lipids and various other biomolecules to their surface upon entering biological fluids, forming an adjustable layer called the protein corona. This phenomenon can largely change the initial physicochemical profile of nanoparticles and further impact their biological identity. They can avoid masking targeting ligands, decrease receptor-binding efficiency, modulate cellular uptake mechanisms, and also promote unintended biodistribution. All of these opsonin proteins (immunoglobulins, complement proteins, fibrinogen and albumin) can enhance recognition by macrophages and speed up clearance from the body via the mononuclear phagocyte system.

Nanoparticle therapeutics are still challenged with huge GMP hurdles for large-scale clinical translation. The manufacture of biomimetic nanocarriers is particularly intricate since it involves biological elements that require cell membranes, exosomes or extracellular vesicles from donor cells. Technical challenges persist in achieving batch-to-batch consistency of membrane composition, protein expression profiles, vesicle purity and biological functionality.

The longevity of BNPs is another issue, particularly in a physiological environment. Due to a variety of physiological conditions that BNPs should experience, including acidic and enzymatic environments in the intestines and complex body fluids such as blood, it is essential for them to have stable properties. If drugs are not stable enough, they may be ineffective and produce unsuitable side effects via their pre-release, nanoparticle agglomeration, or therapeutic payload degradation [98]. The selective material, surface and functionality engineering prevents degradation of the nanoparticles and allows them to be directed toward certain cells based on long-term stability [99]. Furthermore, the integrity of the nanoparticle structure must be maintained during storage and transport in order to facilitate clinical application. This is because degradation or aggregation will change the features of BNPs and thereby abolish therapeutic applications [100].

The biocompatibility of biomimetic nanoparticles remains one of the most challenging and critical aspects of their development, as potential toxicity must be carefully evaluated and addressed before clinical application.

The extent to which BNPs associate with organisms in a manner that renders them nontoxic may be strongly related to their size, charge and composition. In addition, smaller nanoparticles are more likely to be deposited in important organs such as liver and spleen and might eventually result in the development of chronic toxicity [14]. Surface charge and functionalisation are two particularly important features to consider when assessing if nanoparticles interact with immune cells or the cell membrane. For example, without optimization of coating and design, the available nanoparticles may provoke an immunological response which may lead to inflammation, allergy or cytotoxicity [99].

The alteration in body composition of BNPs after repeated dosing is also another issue leading to adverse effects. Nanoparticles that the body’s natural detoxification process, which typically filters out larger particles, cannot dispose of can become a chronic threat to human health by lodging in the bloodstream or getting trapped within crevices between tissues. It is critical in the context of nanoparticles for disease therapies with a long-term perspective (or diseases treatable over prolonged periods) [101]. Second, some of the degradation products of nanoparticles (such as in the case of metals and synthetic polymers) are cytotoxic, which might linger in human cells or tissues [102].

### 5.1. Biomimetic Nanocarriers for Immunotherapy and Gene Therapy in Glioblastoma

The therapeutic potential of biomimetic nanocarriers extends beyond conventional chemotherapeutic delivery to include immunotherapy and gene-based interventions. These approaches are particularly relevant to glioblastoma (GBM), where treatment failure is driven not only by the blood–brain barrier (BBB) but also by an immunosuppressive tumor microenvironment, extensive molecular heterogeneity, infiltrative tumor growth, and the emergence of treatment-resistant cellular populations. Biomimetic nanoparticles may provide a means of simultaneously improving delivery and modulating the biological environment in which these therapies act.

Immune checkpoint pathways represent an important therapeutic target because GBM can establish an immunosuppressive microenvironment that limits effective antitumor immune responses. Immune checkpoint inhibitors directed against pathways such as programmed cell death protein 1 (PD-1)/PD-L1 and cytotoxic T-lymphocyte-associated protein 4 (CTLA-4) aim to restore T-cell activity against tumor cells. However, systemic administration may be limited by inadequate drug accumulation within the brain and by systemic immune-related toxicity. Biomimetic nanocarriers could potentially improve this therapeutic index by transporting checkpoint-modulating agents to the tumor microenvironment and promoting localized release. The current manuscript already describes the potential use of nanoparticles to deliver anti-CTLA-4 and anti-PD-1 antibodies while reducing systemic exposure.

The potential advantage of biomimetic delivery is not restricted to antibody transport. Nanocarriers can be engineered to target tumor cells, tumor-associated immune cells, or other components of the GBM microenvironment, thereby enabling spatially controlled immunomodulation. For example, membrane-derived systems may provide biological interactions that facilitate immune evasion and tumor targeting, whereas ligand-functionalized systems can be directed toward receptors enriched on specific GBM cell populations. Nevertheless, checkpoint inhibition alone may be insufficient because the GBM microenvironment contains multiple immunosuppressive mechanisms. Consequently, biomimetic nanoparticles may be more valuable when checkpoint modulation is integrated with chemotherapy, radiotherapy, vaccination strategies, or other immune-activating approaches.

**siRNA and miRNA delivery.** RNA interference provides an opportunity to suppress genes involved in tumor proliferation, invasion, angiogenesis, treatment resistance, and immune regulation. siRNA can selectively reduce expression of disease-associated genes, whereas miRNAs can regulate multiple target transcripts simultaneously. However, naked RNA molecules are vulnerable to enzymatic degradation and generally exhibit poor cellular uptake. Biomimetic nanocarriers can protect RNA cargo from degradation, improve cellular internalization, and potentially facilitate transport across the BBB. The reviewed literature already identifies biomimetic and exosome-inspired systems as potential carriers for siRNA, miRNA, mRNA, proteins, and chemotherapeutic agents.

Exosome-based and artificial exosome-like systems are particularly attractive for RNA delivery because their biological membrane characteristics may facilitate cellular communication and cargo transfer. The manuscript also identifies artificial exosome nanoparticles containing siRNA/polymer complexes as a strategy for tunable RNA delivery. Nevertheless, the therapeutic application of siRNA and miRNA remains challenging because efficient endosomal escape, intracellular stability, appropriate dose, target specificity, and sustained gene modulation must all be achieved. In addition, miRNA-mediated regulation of multiple transcripts can produce complex biological effects, increasing the importance of careful target validation and off-target assessment.

**CRISPR-based gene editing.** CRISPR-based approaches provide a more direct strategy for modifying disease-associated genes and signaling pathways. In principle, biomimetic nanocarriers can transport CRISPR-associated components, including guide RNA and Cas proteins or appropriate nucleic-acid encoding systems, to tumor cells. Compared with conventional RNA interference, gene editing offers the possibility of producing more persistent genetic modification. The manuscript currently includes CRISPR-loaded biomimetic nanoparticles as an emerging approach for gene editing within the GBM tumor environment.

However, CRISPR delivery presents additional safety and translational challenges. Efficient delivery must occur selectively within tumor cells while minimizing exposure of normal neural cells. Furthermore, heterogeneous target-gene expression means that editing a single molecular pathway may not eliminate all malignant cell populations. Incomplete delivery can leave untreated tumor cells that subsequently contribute to recurrence, whereas unintended editing or prolonged Cas activity could introduce safety concerns. Therefore, future CRISPR-based biomimetic systems should emphasize highly controlled delivery, transient gene-editing activity where appropriate, tumor-specific targeting, comprehensive off-target evaluation, and long-term safety assessment.

**Tumor heterogeneity as a central limitation.** The effectiveness of both immunotherapy and gene therapy is strongly influenced by the molecular and cellular heterogeneity of GBM. The disease contains genetically and phenotypically distinct cellular populations, while alterations involving pathways such as EGFR, PTEN, and TP53 contribute to differences in tumor behavior and therapeutic response. Consequently, a nanocarrier designed against a single receptor or molecular pathway may efficiently target only a subset of tumor cells. Differences in EGFR, transferrin receptor, LRP1, CD44, and other target molecules may also affect nanoparticle uptake between patients and between different regions of the same tumor.

This heterogeneity is particularly important for biomimetic systems because successful targeting depends on the biological characteristics being mimicked or recognized. A carrier optimized for one receptor may lose its targeting advantage when receptor expression is low or absent in another tumor subpopulation. Similarly, variations in BBB integrity and tumor vasculature can influence nanoparticle access to different regions of the tumor. Therefore, molecular profiling and patient stratification may be essential for identifying appropriate targeting ligands and therapeutic cargos.

**Translational challenges and multimodal strategies.** Although biomimetic nanocarriers may address several delivery barriers simultaneously, their application to immunotherapy and gene therapy remains predominantly dependent on achieving reliable tumor-specific delivery and reproducible therapeutic effects. Major challenges include biological heterogeneity, inefficient penetration into infiltrative tumor regions, endosomal trapping of nucleic-acid cargos, off-target activity, immunogenicity, potential toxicity, cargo instability, and variability in nanoparticle composition. In addition, naturally derived biomimetic systems may present manufacturing and characterization difficulties because biological membranes and extracellular vesicles can vary according to their source and isolation procedures. The manuscript already recognizes batch-to-batch variability, scalability, production cost, and regulatory challenges as important barriers for biomimetic nanocarriers.

For these reasons, the most realistic therapeutic strategy may involve multimodal rather than single-agent intervention. Biomimetic nanoparticles could potentially combine immune checkpoint modulation with gene silencing, gene editing, chemotherapy, or radiotherapy in a single treatment framework. Such combinations may simultaneously target distinct mechanisms of tumor survival and reduce the probability that resistant subpopulations escape treatment. However, increasing the number of therapeutic components also increases formulation complexity and regulatory requirements. Thus, future development should balance biological sophistication with manufacturability, reproducibility, safety, and clinically meaningful therapeutic benefit.

Overall, biomimetic nanocarriers provide a promising platform for integrating immunotherapy and gene therapy into GBM treatment. Their greatest potential may lie not simply in transporting more potent therapeutic agents across the BBB, but in enabling spatially controlled, molecularly targeted, and potentially personalized interventions. Nevertheless, successful clinical translation will require validation in heterogeneous and clinically representative GBM models, demonstration of reproducible pharmacokinetics and biodistribution, rigorous safety assessment, and evidence that improved molecular delivery produces meaningful improvements in patient outcomes.

### 5.2. Translational Barriers: Protein Corona, Variability, Manufacturing, Quality Control, and Regulation

Despite the biological advantages of biomimetic nanocarriers, successful translation from experimental models to clinical application remains challenging. The biological identity and therapeutic performance of a nanocarrier can change substantially after administration into the complex physiological environment of the patient. Therefore, evaluation of biomimetic nanocarriers should extend beyond nanoparticle synthesis and in vitro efficacy to include protein interactions, interpatient variability, manufacturing reproducibility, quality control, and regulatory considerations.

**Protein corona formation.** Following systemic administration, nanoparticles rapidly interact with proteins and other biomolecules present in biological fluids, resulting in the formation of a protein corona on the particle surface. This newly acquired biological interface may alter the physicochemical and biological identity of the nanocarrier. In biomimetic systems, such changes are particularly important because the therapeutic function may depend on specific membrane proteins, targeting ligands, or surface characteristics. Adsorption of plasma proteins can mask targeting molecules, modify receptor interactions, influence cellular uptake, alter biodistribution, and affect clearance by the mononuclear phagocyte system. The manuscript already recognizes protein-corona formation as a factor that may modify targeting efficiency and biodistribution. Therefore, future studies should characterize nanoparticle behavior not only in simplified buffer systems but also under physiologically relevant conditions that better represent the biological environment encountered after administration.

**Patient-to-patient variability.** Translation is further complicated by the substantial biological heterogeneity of GBM. Patients may differ in tumor molecular subtype, receptor expression, BBB integrity, vascular density, tumor-associated immune-cell populations, extracellular matrix composition, and treatment history. These differences can influence nanoparticle accumulation, cellular uptake, drug release, and therapeutic response. For example, a biomimetic nanocarrier targeting a receptor that is highly expressed in one patient’s tumor may show limited efficacy in another patient with lower or heterogeneous receptor expression. The molecular heterogeneity of GBM and alterations involving pathways such as EGFR, PTEN, and TP53 already represent important determinants of therapeutic resistance. Consequently, clinical development should incorporate patient stratification and molecular characterization rather than assuming uniform nanoparticle performance across the GBM population.

**Manufacturing reproducibility and scalability.** Manufacturing represents a major translational challenge, particularly for biomimetic systems derived from biological materials. Natural cell membranes and extracellular vesicles may vary according to their biological source, isolation procedure, purification method, storage conditions, and processing parameters. Such variability can influence membrane composition, surface proteins, particle size, drug-loading characteristics, and biological activity. The manuscript currently identifies batch-to-batch variability, complex manufacturing procedures, high production costs, and scalability as important disadvantages of biomimetic systems. At laboratory scale, these variables may be manageable; however, maintaining the same physicochemical and biological characteristics during large-scale production is considerably more difficult. Manufacturing processes must therefore be sufficiently controlled to produce consistent batches with predictable therapeutic performance.

**Quality control and characterization.** Comprehensive quality control is essential for establishing reproducibility and ensuring patient safety. Important quality attributes may include particle size and size distribution, morphology, surface charge, membrane composition, targeting-ligand density, drug or nucleic-acid loading, encapsulation efficiency, release kinetics, structural integrity, sterility, endotoxin levels, and storage stability. For biologically derived biomimetic carriers, additional characterization of membrane proteins, lipid composition, and biological functionality may be required. Importantly, physicochemical characterization should be linked with functional assays so that changes in particle properties can be related to BBB transport, cellular uptake, biodistribution, and therapeutic activity. This is particularly important because relatively small changes in formulation or surface properties can influence biological behavior.

**Regulatory requirements.** The regulatory pathway for biomimetic nanocarriers may be more complex than that for conventional small-molecule drugs because these systems can contain multiple functional components and, in some cases, biologically derived materials. Regulatory evaluation therefore requires clear definition of the composition, manufacturing process, critical quality attributes, impurities, degradation products, stability profile, and biological activity of the final product. Reproducible manufacturing and validated analytical methods are essential for demonstrating that different production batches have comparable quality and therapeutic performance. In addition, preclinical assessment should address biodistribution, pharmacokinetics, toxicity, immunogenicity, and potential accumulation in non-target tissues.

The regulatory complexity may increase further when biomimetic nanocarriers are designed to deliver nucleic acids, gene-editing components, or multiple therapeutic agents. Such multifunctional systems may require simultaneous assessment of the nanocarrier, therapeutic payload, targeting components, and their interactions. Therefore, simplification of nanoparticle architecture, where possible, may improve the feasibility of translation. Designing systems around clearly defined and reproducible components may provide an advantage over highly complex formulations whose individual biological contributions are difficult to characterize.

**Bridging preclinical performance and clinical translation.** Another important issue is the limited ability of conventional preclinical models to reproduce the biological diversity observed in patients. Nanocarriers that demonstrate efficient BBB penetration or tumor growth inhibition in a single animal model may not exhibit equivalent behavior in human GBM. Differences in BBB structure, tumor heterogeneity, immune responses, vascularization, and nanoparticle clearance can influence clinical outcomes. Therefore, translational development should progressively incorporate clinically relevant models and standardized evaluation criteria.

Overall, the clinical translation of biomimetic nanocarriers requires an integrated framework in which biological performance is evaluated together with manufacturing consistency, quality control, safety, and regulatory feasibility. The ability of a nanocarrier to cross the BBB or inhibit tumor growth in an experimental model should therefore not be considered sufficient evidence of clinical readiness. Future development should prioritize reproducible manufacturing, well-defined critical quality attributes, physiologically relevant characterization of protein–nanoparticle interactions, patient stratification, and standardized preclinical-to-clinical evaluation strategies.

### 5.3. Artificial Intelligence-Assisted Design and Personalized Biomimetic Nanomedicine

Artificial intelligence (AI), machine learning (ML), deep learning (DL), and emerging generative approaches are increasingly being explored as computational tools for accelerating the design and optimization of nanomedicine. In the context of biomimetic nanocarriers for glioblastoma (GBM), AI can integrate multidimensional formulation, physicochemical, biological, and therapeutic datasets to identify relationships that may be difficult to establish using conventional trial-and-error approaches. Parameters such as nanoparticle size, surface charge, morphology, composition, ligand density, membrane characteristics, drug-loading efficiency, encapsulation efficiency, and release kinetics can be incorporated into predictive models to identify formulations with desirable delivery and therapeutic characteristics. This approach may reduce experimental iterations and facilitate rational optimization of complex biomimetic systems.

An important emerging application of AI is the prediction of nanoparticle–biological interactions. Following systemic administration, biomimetic nanocarriers interact with plasma proteins, endothelial cells, immune cells, and other components of the biological environment, which can substantially influence their biological identity and fate. Machine-learning models may assist in predicting protein adsorption, protein-corona composition, cellular uptake, biodistribution, clearance, toxicity, and interactions with the blood–brain barrier (BBB). Such predictions are particularly relevant for GBM because successful treatment requires simultaneous optimization of circulation, BBB transport, tumor accumulation, cellular internalization, and therapeutic activity. AI-based models that integrate these variables could therefore support the identification of nanocarrier characteristics associated with improved brain delivery while reducing undesirable systemic interactions.

AI can also contribute to formulation design by linking formulation variables with experimentally measured performance. For example, predictive models can evaluate relationships among carrier composition, surface functionalization, payload characteristics, preparation conditions, particle properties, and drug-release behavior. Optimization algorithms can then prioritize candidate formulations for experimental validation. This is potentially valuable for biomimetic systems because their biological complexity can arise from multiple interdependent variables, including membrane composition, source material, surface proteins, targeting ligands, cargo characteristics, and fabrication conditions. However, computationally optimized formulations must still undergo rigorous experimental characterization because model predictions depend strongly on the quality, diversity, and reproducibility of the datasets used for model development.

Another important opportunity is the application of AI to personalized nanomedicine. GBM exhibits pronounced interpatient and intratumoral heterogeneity, including differences in genetic alterations, receptor expression, cellular composition, vascular characteristics, and tumor microenvironment. The present manuscript already highlights the potential for targeting receptors such as EGFR, transferrin receptor (TfR), LRP1, and CD44 according to tumor characteristics. AI could extend this concept by integrating genomic, transcriptomic, proteomic, imaging, and clinical information to identify patient-specific therapeutic targets and predict the most appropriate nanocarrier characteristics and payload combinations. In this framework, AI-assisted systems could support selection of targeting ligands, therapeutic cargos, carrier composition, and treatment combinations according to the molecular and biological characteristics of individual tumors.

AI may also facilitate adaptive treatment strategies by integrating longitudinal clinical and molecular information. Because GBM can evolve during treatment and develop therapeutic resistance, the optimal nanocarrier formulation or therapeutic payload may not remain constant throughout disease progression. Repeated molecular and imaging data could potentially be incorporated into predictive models to identify changes in target expression, tumor biology, or treatment response and thereby inform subsequent therapeutic strategies. Such approaches are conceptually consistent with the development of customizable biomimetic nanocarriers and precision treatment strategies described in this review.

Despite these opportunities, several limitations currently restrict the clinical implementation of AI-assisted biomimetic nanomedicine. Predictive performance depends on the availability of sufficiently large, high-quality, standardized, and experimentally validated datasets. Differences in nanoparticle synthesis methods, characterization protocols, biological models, experimental conditions, and reporting standards can introduce substantial dataset heterogeneity and limit model generalizability. In addition, complex machine-learning models may lack interpretability, making it difficult to determine why a particular nanocarrier formulation is predicted to perform better than another. Dataset bias, overfitting, insufficient external validation, and differences between preclinical models and human biology represent further challenges. Therefore, AI-generated predictions should be regarded as decision-support tools that require systematic experimental and clinical validation rather than as substitutes for laboratory investigation.

Overall, the integration of AI with biomimetic nanotechnology offers a potentially powerful strategy for moving from empirical nanoparticle development toward data-driven and patient-specific nanocarrier design. For GBM, the greatest value may arise from combining AI-based formulation optimization with molecular profiling, BBB modeling, biological interaction prediction, and longitudinal assessment of treatment response. Nevertheless, successful translation will require standardized datasets, transparent computational workflows, independent validation, and close integration between computational predictions, nanomaterial engineering, preclinical testing, and clinical evaluation. 

## 6. Future Perspectives and Emerging Strategies

Biomimetic nanoparticles have great potential as a stand-alone cancer treatment, but they truly excel when used in combination with other treatments like gene and immunotherapies. By combining those therapies, we can develop a multi-dimensional therapy that no one modality can achieve, and this is exactly what we need to address the complexity and heterogeneity of glioblastoma. Immunotherapy is being used, with some success, to treat just about all types of cancer; it involves harnessing the body’s immune system to fight cancer. Unfortunately, inadequate delivery of immune-activating agents across the BBB has curtailed its application in treating glioblastoma.

One solution is to use biomimetic nanoparticles as delivery vehicles to specifically target certain immune cells in the tumour microenvironment or to enhance the immunogenic triggering of the immune response specifically against glioblastoma cells. For example, nanoparticles can be designed to release anti-CTLA-4 or anti-PD-1 antibodies, which are immune checkpoint inhibitors, into the tumour microenvironment. This can boost the immune response while reducing systemic toxicity [103]. In addition, dendritic cells can be trained to recognise glioblastoma by engineering nanoparticles to transport and target antigens associated with tumours.

Such nanoparticles can be made even more powerful when combined with gene therapy as part of an overall treatment strategy. Gene therapies are in a more literal vein of attack, attempting to repair or replace faulty genes that encourage such glioblastoma cell growth. As for gene like DNA, RNA or siRNA delivery to glioblastoma cells, nanoparticles are a good choice. For instance, therapeutic nanoparticles will deliver therapeutic genes to glioblastoma cells to suppress oncogenes or express tumor-suppressing genes. It is expected that drug resistance models will develop and the recurrence rate will decrease by using BNPs and traditional chemotherapeutic drugs together [104].

The role of artificial intelligence (AI) as a transformational tool to expedite design, optimization and clinical translation of biomimetic nanocarriers and functionalized synthetic nanoparticles is becoming more apparent in recent years. Machine learning (ML), deep learning (DL) and generative AI models can analyze extensive multidimensional datasets for predicting optimal nanoparticle qualities such as particle size, surface charge, shape, ligand density, drug-loading efficiency, release kinetics, blood–brain barrier (BBB) permeation and toxicity profiles. Integrating experimental data with computational modeling allows AI to quickly screen nanoparticle formulations that exhibit favorable therapeutic outputs while minimizing adverse effects. Nanoparticle systems could be personalized according to the molecular signatures of patients. As a consequence, EGFR-, TfR-, LRP1- or CD44-overexpressing tumors can be specifically targeted with the respective functionalized nanoparticles. Another approach is developing personalized biomimetic nanocarriers by utilizing autologous cell membranes or patient-derived exosomes which may lead to reduced immunogenicity and increased specificity of targeting.

Another area that is getting a lot of attention is radiation and nanotechnology. The effectiveness of radiation therapy can be enhanced by nanoparticles, which function to sensitize cancer cells towards radiation and local mediums that release reactive oxygen species (ROS) upon radiation exposure, leading to greater DNA damage in such cancer cells. A novel approach to treatment of glioblastoma involved therapy using nanoparticles with immunotherapy, gene therapy and radiation. This comprehensive multimodal therapy is intended to optimize effect while limiting toxicity.

The future of glioblastoma treatment is also tied to personalized medicine, for which patients are treated based on individual traits. Heterogeneity in genetic mutations, epigenetic changes and the microenvironment is a hallmark of GBM cells. Therefore, individualized therapeutic strategies are needed, as a single treatment approach is unlikely to be effective for all patients. The potential of treating patients with tailor-made treatments can only be achieved by highly customisable nanoparticles such as those presented here.

The treatment regimens for those with glioblastoma can be fine-tuned by pairing nanoparticles with molecular profiling methods. By examining the genetic mutations, protein expressions and other hallmarks contained in each patient’s tumour, doctors can zero in on the most promising treatment targets with molecular profiling. Nanoparticles surface-modified with these receptor ligands could be used to deliver the drug selectively in cancer patients who have tumours that overexpress certain receptors such as epidermal growth factor receptor (EGFR) [105]. Being able to manipulate nanoparticles (whether they are bearing chemo, gene or immunotherapeutic payloads) means personalised treatment strategies based on evolving tumour features over time.

In addition, advances in AI and machine learning will power personalised glioblastoma therapies. An AI-based approach will enable treatment selection by computing on large datasets including proteomic, genomic and clinical data on a patient level. Apart from modelling BNPs, this will allow online adaptations of treatment plans with the aim of maximizing efficacy and minimizing side effects [106].

One of the major obstacles to treating glioblastoma is the development of resistance; however, this risk can be mitigated using personalised therapy [107]. One way to circumvent these obstacles is by engineering nanoparticles to target and recognise specific chemical pathways that promote tumour growth and resistance. Patients with glioblastoma may have better long-term prognoses and survival rates because of this targeted selectivity.

Personalized treatment, biomimetic multifunctional nanoparticles and advanced technology nanomaterials are going to be the future weapons against several diseases [108,109]. Advances in nanoparticle design have enabled combination therapy for glioblastoma through the development of stimuli-responsive and multifunctional particles that facilitate precise and effective drug delivery. Second, a multimodal strategy to tackle the complexity of glioblastoma and enable more powerful therapeutic benefits can be achieved through immunotherapy, gene therapy, and radiotherapy via biomimetic nanoparticles. Third, the treatment of glioblastoma may be transformed by personalised or precision medicine that combines molecular profiling with AI. That would make it possible to develop drugs tailored specifically for the individual characteristics of each patient’s tumour. Thanks to relentless innovation, the field of biomimetic nanoparticles is now taking unprecedented strides in its fight against cancer and other hard diseases.

The successful clinical translation of biomimetic nanocarriers (BNCs) for glioblastoma (GBM) will require paradigm shifts in experimental modeling, personalized medicine, and large-scale manufacturing [110]. To realize the full potential of these therapies, future research must prioritize several critical emerging directions:

- **Advanced Preclinical Models:** Standard two-dimensional cultures strip away the blood–brain barrier (BBB) and vascular heterogeneity that govern therapeutic responses in vivo. Future research must prioritize dynamic microfluidic BBB-on-a-chip platforms and patient-derived brain organoids. These advanced models accurately simulate the tight junction dynamics of the BBB and preserve the spatial architecture of the tumor microenvironment, providing a more reliable assessment of nanomedicine efficacy prior to in vivo testing.
- **Multifunctional Theranostic Platforms:** BNCs are uniquely positioned for theranostic applications, which combine diagnostic imaging and targeted therapy within a single nanocarrier framework. By integrating contrast agents, radioactive isotopes, or fluorescent probes with therapeutic payloads, these platforms enable the real-time monitoring of BBB penetration, precise tumor localization, and the in vivo assessment of drug release kinetics.
- **Personalized Nanomedicine:** Given the profound inter-patient and intra-tumoral heterogeneity of GBM, one-size-fits-all drug delivery systems are often insufficient]. Future strategies will leverage genomic and proteomic profiling to customize biomimetic surface modifications. For instance, engineering BNCs with patient-derived tumor cell membranes could facilitate the development of personalized anticancer vaccines or highly specific targeted therapies tailored to an individual’s unique receptor expression profile.
- **Improving Clinical Translation:** To bridge the gap from bench to bedside, researchers must overcome formidable manufacturing challenges. This necessitates the development of scalable, reproducible fabrication protocols that maintain stringent batch-to-batch consistency without compromising the functional integrity of biomimetic coatings. Furthermore, establishing standardized regulatory frameworks to evaluate the long-term biosafety, immunogenicity, and pharmacokinetic profiles of complex biological nanocarriers will be essential for accelerating clinical approval.

## 7. Conclusions

For aggressive, highly complex cancers such as GBM, BNCs offer a highly promising approach for targeted therapeutic delivery. These bioinspired nanocarriers address many limitations of traditional drug delivery systems by selectively transporting therapeutic agents to cancer cells while sparing healthy neural tissue. Because glioblastoma cells frequently overexpress surface receptors such as EGFR and the transferrin receptor (TfR), BNCs can be functionalized to exploit these pathways for efficient, localized drug delivery. Furthermore, by optimizing the size, surface charge, and biomimetic coating of these nanocarriers, researchers have developed strategies to facilitate transport across the blood–brain barrier. The integration of BNCs into combination therapy regimens represents a critical advancement in glioblastoma management, as these platforms can co-deliver a diverse array of payloads, including chemotherapeutic drugs, immune checkpoint inhibitors, and gene-editing reagents (e.g., CRISPR or RNA interference). Ultimately, navigating the translational challenges of BNCs—particularly large-scale manufacturing and in vivo stability—will be essential to realizing their potential in clinical oncology.

Overall, the distinctive contribution of this review lies in connecting the biological rationale of biomimetic nanocarrier design with the major therapeutic and translational barriers of glioblastoma. By considering BBB/brain–tumor barrier transport, biological targeting, immunotherapy, gene delivery, preclinical evidence, and clinical translation within a single framework, the review highlights not only what biomimetic nanocarriers can achieve but also where current evidence remains insufficient. This perspective emphasizes that successful translation will require demonstration of reproducible biological targeting, effective penetration of heterogeneous and infiltrative GBM, long-term safety, scalable manufacturing, and clinically meaningful therapeutic benefit rather than improved nanoparticle accumulation alone.

## Data Availability

All data supporting the findings of this study are available within the paper.
